# Distributed Lag Analyses of Daily Hospital Admissions and Source-Apportioned Fine Particle Air Pollution

**DOI:** 10.1289/ehp.1002638

**Published:** 2010-12-20

**Authors:** Ramona Lall, Kazuhiko Ito, George D. Thurston

**Affiliations:** New York University, School of Medicine, Department of Environmental Medicine, Tuxedo, New York, USA

**Keywords:** cardiovascular, distributed lag model, generalized linear models, hospital admissions, New York City, particulate matter, positive matrix factorization, respiratory, source apportionment, time series, trace element species, traffic

## Abstract

**Background:**

Past time-series studies of the health effects of fine particulate matter [aerodynamic diameter ≤ 2.5 μm (PM_2.5_)] have used chemically nonspecific PM_2.5_ mass. However, PM_2.5_ is known to vary in chemical composition with source, and health impacts may vary accordingly.

**Objective:**

We tested the association between source-specific daily PM_2.5_ mass and hospital admissions in a time-series investigation that considered both single-lag and distributed-lag models.

**Methods:**

Daily PM_2.5_ speciation measurements collected in midtown Manhattan were analyzed via positive matrix factorization source apportionment. Daily and distributed-lag generalized linear models of Medicare respiratory and cardiovascular hospital admissions during 2001–2002 considered PM_2.5_ mass and PM_2.5_ from five sources: transported sulfate, residual oil, traffic, steel metal works, and soil.

**Results:**

Source-related PM_2.5_ (specifically steel and traffic) was significantly associated with hospital admissions but not with total PM_2.5_ mass. Steel metal works–related PM_2.5_ was associated with respiratory admissions for multiple-lag days, especially during the cleanup efforts at the World Trade Center. Traffic-related PM_2.5_ was consistently associated with same-day cardiovascular admissions across disease-specific subcategories. PM_2.5_ constituents associated with each source (e.g., elemental carbon with traffic) were likewise associated with admissions in a consistent manner. Mean effects of distributed-lag models were significantly greater than were maximum single-day effect models for both steel- and traffic-related PM_2.5_.

**Conclusions:**

Past analyses that have considered only PM_2.5_ mass or only maximum single-day lag effects have likely underestimated PM_2.5_ health effects by not considering source-specific and distributed-lag effects. Differing lag structures and disease specificity observed for steel-related versus traffic-related PM_2.5_ raise the possibility of distinct mechanistic pathways of health effects for particles of differing chemical composition.

Presently, there is a knowledge gap regarding the possible role of particulate matter (PM) chemical composition in explaining the adverse health effect associations that have been found with PM mass. Multicity time-series studies suggest that PM effects are not homogeneous across cities; rather, the results appear to be driven by specific regions (e.g., U.S. Northeast) and seasons (spring and summer) (Dominici et al. 2002, 2006; Franklin et al. 2008; Peng et al. 2005). To better understand the role of composition, several recent multicity studies have examined whether variability in proportions of PM constituents across U.S. cities can explain the observed variations in relative risks (RRs) of PM and mortality or morbidity outcomes. These studies point to certain PM constituents: nickel (Ni), vanadium (V), elemental carbon (EC), aluminum, arsenic, sulfates, and silica (Si) (Bell et al. 2009; Franklin et al. 2008; Lippmann et al. 2006; Zanobetti et al. 2009). Dominici et al. (2007) have also shown that the effect modification by Ni and V in the National Morbidity, Mortality, and Air Pollution Study is weakened by the removal of certain New York counties. The two-stage analyses conducted in these multicity studies explain the effect modification by PM constituents, but do not directly test for an association between these constituents and mortality and morbidity outcomes. Nonetheless, findings from these multicity studies warrant further epidemiological investigations that directly test for an association between PM sources or constituents and health in major cities, especially in the U.S. Northeast.

Considering the case of New York City (NYC), we used time-series analysis to investigate the effects of daily source-related fine PM [aerodynamic diameter ≤ 2.5 (PM_2.5_)] on older adults who were admitted to hospitals for respiratory and cardiovascular causes during 2001 through 2002. NYC has a population of approximately 8 million persons; of these, 11.7% are ≥ 65 years of age and eligible for Medicare (U.S. Census Bureau 2000). This represents a large population group, with a significant number of hospital admissions on a daily basis.

The U.S. Environmental Protection Agency (EPA) Chemical Speciation Network (CSN) conducts sampling of pollutants every third day in NYC. Because of this sampling schedule, researchers who use these data in a time-series study require longer study periods. In addition, this sampling process prevents investigations of possible distributed-lag associations, such as individual day effects versus cumulative mulitday effects.

In the present study, we used speciation data collected by New York University (NYU) on a daily basis during 2001 through 2002. This sampling schedule provided greater power and allowed the investigation of distributed lag effects that were not possible in past analyses.

## Materials and Methods

### Sampling site details

In 2001, NYU set up a monitoring site in Manhattan (NYC) to collect daily PM_2.5_ filter samples for subsequent chemical analyses. The site was centrally situated on a second-floor rooftop of the Hunter College Public Health Building on the East Side of Manhattan [at 27th St. and 1st Ave.; see Supplemental Material, Figure 1 (doi:10.1289/ehp.1002638)]. The NYU sampling site was located one avenue block (~ 0.13 miles) west of the Franklin D. Roosevelt (East River) Drive, a major artery of traffic through Manhattan, and within the 8.5 square miles of the “Central Business District” identified as being the “primary generator of congestion in the five boroughs” (Partnership for New York City 2006).

The samples that were collected on a daily basis from 1 January 2001 through 31 December 2002 were analyzed in this study.

### Daily trace element and EC data

PM_2.5_ mass concentration (by filter weighing), trace element concentrations (by X-ray fluorescence technique), and black carbon measurements (using the reflectance technique) were conducted at the NYU Nelson Institute of Environmental Medicine laboratory (Tuxedo, NY). Additional details regarding sample collection and analysis are provided in Lall and Thurston (2006).

During the first year of operation, direct measurements of EC concentrations from a carbon analyzer (5400 Ambient Particulate Carbon Monitor, Rupprecht & Patashnick Co., Albany, NY) were also available at the monitoring site; we used these to derive the EC calibration curve for the absorption coefficients estimated via the reflectance technique.

### Medicare hospital admissions daily count data

The time-series health analyses were conducted using Medicare hospital admissions data for the older adult population (≥ 65 years of age) in NYC during 2001 through 2002. The categorization of the admissions data was based on codes from the *International Classification of Diseases*, revision 9 (ICD-9) (World Health Organization 1977). We used SAS software (version 9.1; SAS Institute Inc., Cary, NC) to compute total daily admissions by respiratory and cardiovascular disease categories. Counts were restricted to emergency and urgent admissions of those residing in one of the five NYC boroughs (Manhattan, Brooklyn, Queens, Bronx, and Staten Island).

To be consistent with previous time-series studies (U.S. EPA 2004), we used the following ICD-9 codes: total respiratory hospital admissions included pneumonia (480–486), chronic obstructive pulmonary disease (COPD; 490–492 and 496), acute bronchitis and bronchiolitis (466), and asthma (493); total cardiovascular hospital admissions included dysrhythmia (427), ischemic heart disease (IHD; 410–414), heart failure (428), and stroke (431–437).

### Time-series generalized linear model

Using a generalized linear model, we conducted time-series analyses of daily hospital admissions and source-related PM_2.5_; we controlled for season, wintertime influenza episode, weather, day of the week, and other possible confounders (e.g., federal holidays). To model seasonal trends and remove autocorrelation in the time-series data, we used a natural cubic spline of date with 9 degrees of freedom per year, based on assessing best model fit using the Akaike information criterion and residual autocorrelation. Same-day temperature (heat) and 2- to 3-day lagged temperature (cold) effects were also modeled using natural cubic splines of daily temperature, with 3 degrees of freedom. An additional term, “hot and humid” days (temperature > 78°F and relative humidity > 96%), was included to account for possible confounding by an interaction of high temperature and high humidity not explained by the cubic spline smooth of temperature.

PM_2.5_ mass, apportioned into various source categories using positive matrix factorization (PMF), was then included into the model one at a time. For a summary of the concentration estimates of the trace PM_2.5_ constituents considered in the PMF source apportionment analysis, see Supplemental Material, Table 1 (doi:10.1289/ehp.1002638). The PMF source apportionment model is also described in the Supplemental Material (doi:10.1289/ehp.1002638). Table 1 shows the 5th–95th percentile differences for each source; these increments were used to estimate RRs and 95% confidence intervals (CIs).

Given the relatively short time period considered by the study analysis, and the statistical concerns regarding multiple comparisons, we focused on two categories—total respiratory and total cardiovascular—to maximize available power. Lags of 0–3 days of exposure were considered in these models to investigate the possible time distribution of effects. We evaluated the pollution variable at lags 0 through 3 days based on previous studies. In particular, the largest U.S. multicity study that examined the associations between PM_2.5_ and older adult hospitalizations, which evaluated lags 0 through 2 days, found the strongest associations for cardiovascular and respiratory categories mostly at lag 0 days but at lag 2 days for some outcomes such as IHD and respiratory tract infection (Dominici et al. 2006). Therefore, we examined lags up to 3 days.

PM_2.5_ trace element constituents, considered as “key” tracers of the identified sources, were also included individually in a separate analysis in order to validate findings for source-apportioned mass. Positive associations between source-specific PM_2.5_ and respiratory or cardiovascular health outcome were also investigated by their specific disease subcategory, to check for consistency across specific disease outcomes and specific disease categories driving the association. Finally, as part of a sensitivity analysis, time-series models for seasons (i.e., summer, from May to October vs. winter, from November to April) and by borough (e.g., admission counts for Manhattan only) were also investigated.

Additionally, for cases with observed source PM–health associations, we applied an unconstrained distributed lag model to assess whether the cumulative effects (over the 4 lag days being considered) were greater than the effects for the single highest-day lag. This approach also allowed us to compare the results of the present study with those from past analyses that used the maximum lag approach. In the distributed lag model, instead of including only the single highest-day–lag pollution, multiple lagged days of pollution were simultaneously included in the time-series model. Distributed lag models have been previously used in air pollution studies to estimate the overall effect of exposure on health outcome (e.g., Braga et al. 2001; Schwartz 2000; Wyzga 1978). The issue of potential underestimation of effects using single-lag estimates has been frequently discussed in the epidemiological community in the past (Goodman et al. 2004; Roberts 2005; Zanobetti et al. 2003).

## Results

### NYC PM_2.5_ exposure assessment

A detailed description of the source apportionment results is presented in the Supplemental Material (doi:10.1289/ehp.1002638). Briefly, based upon the trace metal associations with each PMF component, we identified seven “source” categories: long-range transported sulfates, traffic, residual oil, steel metal works dust, soil, World Trade Center (WTC) plume, and wintertime chlorine peaks. We considered five of these seven PM_2.5_ sources *a priori* for subsequent health analyses; we excluded the WTC plume and wintertime chlorine from the health analysis because these were rare events with only isolated spikes. Given the timing of the highest steel particle source impacts, the steel “source” category clearly includes particles arising from the WTC cleanup metal works operations in 2001–2002 (e.g., from cutting and welding) but may also include other similar construction and demolition activities in the city. We also similarly identified the five sources being considered in the previous analysis using data for 2001 alone (Lall and Thurston 2006) and by other studies conducted in NYC (Ito et al. 2004). We chose elemental or chemical “tracers” of each of these sources: sulfur (S; long-range transport sulfates), EC (traffic), Ni (residual oil), manganese (Mn; steel), and Si (soil). Table 1 summarizes source-related PM_2.5_ contributions.

### Time-series health analyses of PM_2.5_ and source-apportioned PM_2.5_

During the study period, a total of 31,302 hospital admissions for respiratory-related illnesses and 72,415 admissions for cardiovascular-related events were found in the Medicare admissions data among NYC adults ≥ 65 years of age (Table 2). The daily mean estimates were 43.9 for respiratory- and 101.6 for cardiovascular-related hospital admissions.

### Respiratory hospital admissions

**Figure 1:**
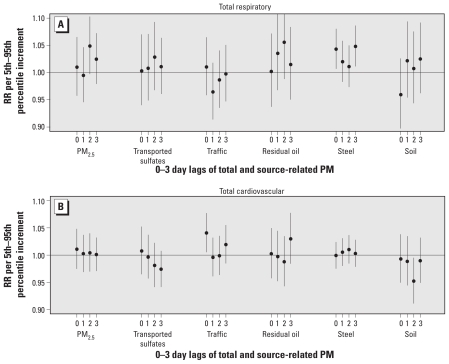
RRs for total respiratory (*A*) and total cardiovascular (*B*) admissions per 5th–95th percentile increment in PM_2.5_ mass and source-related PM.

**Figure 2:**
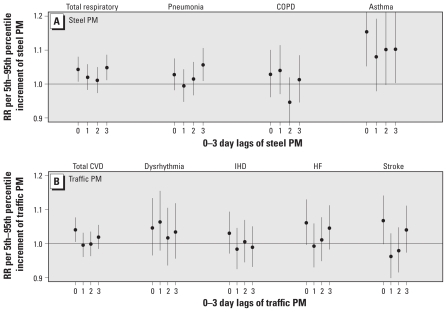
RRs for disease-specific respiratory hospital admissions per 5th–95th percentile increment of steel PM (*A*) and disease-specific cardiovascular hospital admissions per 5th–95th percentile increment of traffic-related PM (*B*).

Total respiratory hospital admissions were associated with nonspecific PM_2.5_ at lag 2 day (Figure 1A). However, in further analyses, 2-day PM_2.5_ lags were not consistently associated with subcategories of specific respiratory outcomes. Among the five source categories, PM_2.5_ from steel (increment = 2.1 μg/m^3^) was associated with same-day lag (RR = 1.043; 95% CI, 1.007–1.080) and 3-day lag (RR = 1.048; 95% CI, 1.011–1.086) for respiratory admissions. We also found positive and significant associations for this source category with the two respiratory subcategories: pneumonia and asthma (Figure 2A).

### Sensitivity analysis of steel metal works PM versus respiratory outcomes

We also considered multiple PM_2.5_ source categories simultaneously in time-series models and investigated each NYC borough separately. The association between the steel dust and respiratory admissions was not affected by the simultaneous inclusion of other sources in the model. Because the steel metal work related to cleanup activities at the WTC site (located at the southernmost tip of Manhattan) appears to be the primary source of the pollution in this observed association, we also conducted the health analyses for total respiratory hospital counts by borough of residence to test if Manhattan was driving the association. We found significant associations for Manhattan on lag 0 and lag 3 and for the Bronx on lag 0. We found a positive association in Brooklyn for same-day exposures to this source but no such associations for the more distant boroughs, Staten Island and Queens. Thus, the strongest PM–health association for this steel-related particle source was for the Borough of Manhattan, as would be expected if the origin of the pollution were the WTC site. However, it should be noted that this association is being driven by a limited number of days during spring 2002 with high concentrations from this source. Season-specific analyses did not indicate any clear differences by season. Excluding data for the period between September and December 2001 (i.e., immediately after the attack on the WTC and during the initial cleanup efforts) from the analysis also did not change the results, indicating a robustness of associations by this type of steel-related particle beyond that derived from the initial WTC cleanup.

### Cardiovascular hospital admissions

Total PM_2.5_ mass and PM_2.5_ from sources other than traffic-related were not associated with total cardiovascular hospital admissions (Figure 1B). However, same-day traffic-related PM_2.5_ exposures were positively associated with total cardiovascular admissions (RR = 1.041; 95% CI, 1.005–1.077; for a 2.8 μg/m^3^ increment in PM_2.5_ from traffic). Same-day traffic effects were also consistently associated with the subcategories of cardiovascular disease: stroke and heart failure (Figure 2B). Although IHD and dysrhythmia were not significantly associated, they, too, were positively correlated with same-day traffic exposures. Given the consistency in the observed associations between same-day exposure and cardiovascular admissions, these results appear to suggest immediate effects as a result of exposure to traffic-related PM.

### Sensitivity analysis of traffic PM versus cardiovascular outcomes

Our results were not affected by the simultaneous inclusion of the other sources in the time-series model. We observed especially high concentrations from the traffic source (> 4 μg/m^3^) on a few models. Removing these high days from the analysis did not change the positive association found for cardiovascular disease, stroke, and heart failure, with stroke remaining significant. We found same-day traffic effects on cardiovascular admissions for both “colder” (November–April) and “warmer” (May–October) months (data not shown); however, only wintertime traffic was statistically significant. NYC borough-specific associations were not obvious for traffic PM_2.5_, unlike the steel dust versus respiratory associations, as expected for this citywide source category.

### Distributed lag model

**Figure 3:**
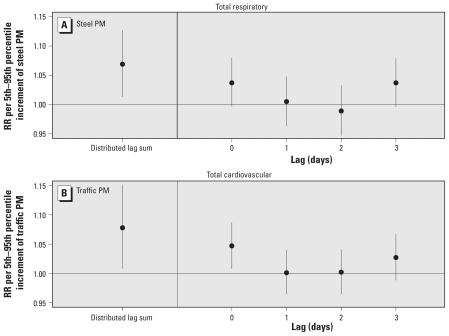
Distributed lag sum and simultaneous inclusion of 0- to 3-day lags for steel-related PM and total respiratory hospital admissions (*A*) and for traffic-related PM and total cardiovascular hospital admissions (*B*).

Because of the novel availability of daily mass contributions in these data, we further investigated the cumulative effect of the steel and traffic sources using an unconstrained distributed-lag model, which allows multiple lag days of pollution to be simultaneously included in the time-series model. An unconstrained distributed lag model of total respiratory admissions considered over a consecutive 4-day period provided cumulative risk estimates for steel PM that were higher than risk estimates for any single day: RR = 1.069 (95% CI, 1.013–1.127) versus RR = 1.043 (95% CI, 1.007–1.080; Figure 3A). Similarly, the cumulative traffic-related cardiovascular effects over 0–3 days (RR = 1.078; 95% CI, 1.008–1.152) were much higher than those observed in the single maximum same-day lag model (RR = 1.041; 95% CI, 1.005–1.077; Figure 3B). Thus, these results show that, in this case, consideration of only a single-day concentration consistently underestimated the total distributed-lag effects of source-related PM_2.5_.

### Sensitivity analysis of model specifications and exposure metric

We also investigated sensitivity of results to model specifications. The positive associations described above were robust to changes in model specifications for both weather (i.e., degrees of freedom used to model seasonality) and temperature (e.g., quintile indicators instead of natural cubic splines).

**Figure 4:**
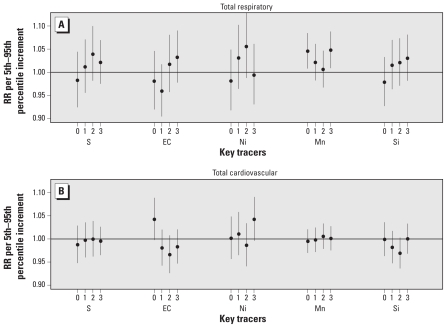
RRs for total respiratory-related (*A*) and total cardiovascular-related (*B*) hospital admissions per 5th–95th percentile increment for trace element PM_2.5_ constituents.

Using key elemental tracers (Mn and EC), rather than source-related mass contributions (steel and traffic) obtained from source apportionment analyses, provided similar findings. That is, Mn was associated with respiratory admissions for same-day lag (RR = 1.046; 95% CI, 1.008–1.085) and 3-day lag (RR = 1.048; 95% CI, 1.009–1.088), and same-day EC was associated with cardiovascular admissions (RR = 1.042; 95% CI, 0.998–1.089; Figure 4).

## Discussion

Among the numerous published time-series studies of PM and mortality or morbidity outcomes, very few studies have considered either source-apportioned mass or the distributed lag of these associations, and to our knowledge none have previously considered both. Although the U.S. EPA CSN provides a new opportunity to conduct studies to further elucidate the role of specific sources, the unique advantage of the NYC daily data set used in this study is the ability to test the distributed lag structure of the source-apportioned PM and health effects over multiple days. More studies investigating source apportioned PM_2.5_ mass or PM_2.5_ constituents are necessary to add to our current understanding of PM-related health effects.

Of the few epidemiological studies that have used source-apportioned mass exposures to study the PM–health effects associations, most have considered mortality outcomes (Ito et al. 2006; Laden et al. 2000; Mar et al. 2000, 2006; Ostro et al. 2007; Thurston et al. 2005; Tsai et al. 2000). Consistently, across these studies, mortality associations have been found with combustion-related particles, particularly for sulfates, coal combustion, and traffic. In a more recent study of hospital ED visits conducted in Atlanta, Georgia, Sarnat et al. (2008) found associations between PM_2.5_ from mobile sources and biomass burning with both cardiovascular and respiratory ED visits, and between sulfate-rich secondary PM_2.5_ and respiratory visits.

In the present study, we did not consistently observe a significant association between nonspecific PM_2.5_ mass or sulfate PM and hospital admissions, as has been found in most previously published studies. This could be attributed to the shorter study period of 2 years available for this study. However, in contrast, PM_2.5_ mass contributions from certain source categories (i.e., steel- and traffic-related) were strongly associated with hospital admissions. In particular, the steel metal works PM_2.5_ source was associated with respiratory admissions on multiple days, whereas the traffic PM_2.5_ source was associated with same-day cardiovascular outcomes. The specificity of disease outcome associated with the two source categories, and the different lag structures observed for each would suggest the possibility of distinct mechanistic pathways for particles with differing chemical composition that would not be discerned when considering total PM_2.5_ mass as the index of exposure.

The steel source category reflects construction activities in and around the NYC area and is especially elevated during September 2001 and May 2002, a period associated with the WTC fires and cleanup metal works activities at Ground Zero (< 3 miles from the NYU monitoring site). The associations found for steel and respiratory admissions in this analysis are driven by a limited number of days of high-source-impact concentrations during May 2002. However, we also observed this association for specific respiratory disease subcategories (particularly asthma-related admissions) and for the city-central boroughs of Manhattan, Bronx, and Brooklyn (but not for most distant Queens or Staten Island). Pollen count data were not available to test for possible confounding, but if pollen were the underlying cause of the associations found, one would expect to see associations with respiratory admissions fairly uniformly across the boroughs, which we did not. Finally, there is some biological plausibility to this newly observed steel PM–hospital admissions association, given the similar findings in the Utah steel mill studies. Pope (1989) found PM_10_ (including major contributions from a local steel mill) to be most strongly correlated with hospital admissions for bronchitis and asthma (similar to disease specificity found for steel PM in the present study). Also, in a toxicology study, particles collected before and after the Utah steel mill closure were instilled into the lungs of human volunteers, and greater inflammatory responses were found for PM collected during the operation of the steel mill versus after the closure of the mill (Ghio and Devlin 2001).

Traffic-related PM_2.5_ was strongly and consistently associated with same-day cardiovascular admissions, when considering disease-specific categories (e.g., stroke and heart failure) or season (e.g., winter). This is coherent with other time-series studies investigating source-apportioned mass and mortality or morbidity outcomes. Researchers are investigating mechanisms that are related to onset of cardiovascular-related symptoms within a few hours of traffic exposures (e.g., Lanki et al. 2006; Peters et al. 2004; Schwartz et al. 2005). Thus, the associations we found in the present study are consistent with the results from other recent findings that traffic pollution is associated with cardiovascular disease impacts. Further, like other published results of cumulative effects of total PM, in the present study we also found that the cumulative impact of traffic-related PM_2.5_ was greater than any single lag considered, suggesting that the consideration of any single lag, even the maximum lag day, tends to underestimate the total distributed-lag health effects.

PM_2.5_ trace constituents provided similar and consistent associations with hospital admissions, as did associated source-apportioned mass categories, suggesting the equivalence of either of these approaches once the overall tracer–source component relationships have been identified via multivariate methods, such as factor analysis or PMF.

Time-series studies are generally limited to the use of central monitoring sites to estimate population exposures. In the case of NYC, comparison across multiple monitoring sites within the city has shown that whereas PM_2.5_ and secondary sulfates are spatially homogeneous, constituents of local combustion are not as highly correlated spatially (Ito et al. 2004). Similarly, in the present study, certain PM_2.5_ constituents were more spatially homogeneous (e.g., sulfur, Ni) than were others (e.g., Mn) when comparing concentrations at NYU versus U.S. EPA CSN sites in NYC (Table 3). The NYU sampling site was located within the Central Business District, which has been shown to be the “primary generator of congestion in the five boroughs” (Partnership for New York City 2006). Therefore, even though this study uses only a single monitoring site, the location of this particular site is thought to be a reliable indicator of day-to-day exposure patterns for a wider area throughout NYC. It is not possible to fully assess whether this particular site provides better representation of population exposures to certain local sources (e.g., traffic) compared with other local sources (e.g., residual oil). However, despite this limitation of using a single site, our study, as well as other time-series studies of source-apportioned mass and health, also showed consistent traffic-related PM associations with cardiac health, as discussed above.

Finally, this study shows that considering just the nonspecific PM_2.5_ mass or the maximum day lag of source-specific effects significantly underestimates the public health impacts of PM air pollution in this case. For example, application of the RRs derived in this study to NYC hospital admissions data estimates that some 240 annual cardiovascular-related hospital admissions are attributable to average PM_2.5_ mass concentration (17.1 μg/m^3^) observed for NYC. In comparison, applying the source-specific results indicate that more than 600 cardiovascular-related hospital admissions are attributable to average traffic-related PM_2.5_ (1.2 μg/m^3^). This reflects a more than 2-fold difference in attributable risk when considering nonspecific PM_2.5_ versus source-specific PM_2.5_. Furthermore, results from the distributed-lag model for source-apportioned steel and traffic mass show cumulative effects to be much larger, compared with effects observed on a single day (e.g., roughly double in the case of traffic). These results therefore suggest that, by considering only a maximum single-day lag, and only for PM_2.5_ mass, past studies may well have greatly underestimated the true total acute effects of PM air pollution.

## Conclusions

Findings from our study suggest that toxicity of PM_2.5_ depends on the source (and, therefore, the composition) of that pollution and that considering nonspecific PM_2.5_ mass alone may greatly underestimate the public health impacts of PM_2.5_ air pollution. Cumulative effects of source-related PM_2.5_ distributed over several days were also significantly greater than the effect size reported for any single-day lag, indicating further underestimation of air pollution effects by studies that rely on a single maximum-day lag. The specificity of the respiratory and cardiovascular disease outcomes associated with steel- and traffic-related PM_2.5_, respectively, as well as the different lag structures observed for these associations, would suggest potentially different mechanistic pathways for each of these compositionally different PM_2.5_ sources. This work indicates that the current PM_2.5_ mass-based standard might not be sufficiently protective of public health and that estimates of the health benefits of reducing PM_2.5_ air pollution may well have been significantly underestimated in past PM_2.5_ mass-based health effects analyses.

## Figures and Tables

**Table 1:** Mean ± SD source contributions (μg/m^3^) for overall (2001–2002) and year and for overall 5th–95th percentile differences in source contributions (μg/m^3^).

Source category	Overall	2001	2002	5th–95th percentile difference
Transported sulfates	9.7 ± 8.0	9.4 ± 7.6	10.0 ± 8.4	25.5
Residual oil	3.6 ± 2.4	3.7 ± 2.5	3.5 ± 2.3	7.4
Traffic	1.2 ± 0.9	1.2 ± 0.8	1.1 ± 1.1	2.8
Soil	0.7 ± 0.9	0.5 ± 0.6	1.0 ± 1.1	2.8
Steel metal works	0.6 ± 0.9	0.4 ± 0.7	0.7 ± 1.0	2.1

**Table 2:** Daily average and total counts of hospital admissions for respiratory and cardiovascular causes in NYC (2001–2002), by disease categories and borough.

Admission category	Daily average	Total (2001–2002)
Cause-specific admissions		
Respiratory	43.9	31,302
Pneumonia	26.4	18,811
COPD	11.1	7,894
Asthma	5.1	3,644
Cardiovascular	101.6	72,415
Dysrhythmia	14.4	10,270
IHD	30.8	21,973
Heart failure	27.8	19,822
Stroke	20.3	14,472
Admissions by NYC borough		
Respiratory	43.9	31,302
Manhattan	9.3	6,624
Brooklyn	13.1	9,359
Bronx	7.8	5,534
Queens	11.4	8,156
Staten Island	2.3	1,629
Cardiovascular	101.6	72,415
Manhattan	20.3	14,440
Brooklyn	32.8	23,384
Bronx	16.4	11,690
Queens	27.4	19,504
Staten Island	4.8	3,397

**Table 3:** Correlation (*r*) between day-to-day observations at the NYU site versus three U.S. EPA CSN sites in NYC.

Site (no. of observations)	EC	Ni	Mn	Si	S
U.S. EPA CSN sites					
New York Botanic Garden (*n* = 181)	0.53	0.44	0.14	0.66	0.95
Intermediate School 52 (*n* = 161)	0.39	0.62	0.18	0.72	0.93
Queens College (*n* = 120)	0.50	0.61	0.12	0.12	0.96
